# B10 cell-induced PD-L1/PD-1-linked macrophage polarization in periodontitis

**DOI:** 10.1515/jtim-2025-0038

**Published:** 2025-07-24

**Authors:** Linxi Zhou, Mingyue Fan, Lunguo Xia, Yuntian Chen, Xiaozhe Han, Bing Fang

**Affiliations:** Department of Orthodontics, College of Stomatology, Shanghai Jiao Tong University School of Medicine Affiliated Ninth People's Hospital, Shanghai, China;; National Clinical Research Center for Oral Diseases, Shanghai Key Laboratory of Stomatology & Shanghai Research Institute of Stomatology, Shanghai, China;; Department of Immunology and Infectious Diseases, The Forsyth Institute, Cambridge, MA, USA; Department of Orthodontics, Shanghai Xuhui District Dental Center, Shanghai, China;; Department of Respiratory and Critical Care Medicine, Shanghai Jiao Tong University School of Medicine Affiliated Ninth People's Hospital, Shanghai, China;; Department of Oral Science and Translation Research, Nova Southeastern University College of Dental Medicine, Fort Lauderdale, FL, USA

**Keywords:** regulatory B cells, macrophage polarization, PD-L1/PD-1 signalling pathway, periodontal disease, immunomodulation, inflammation

## Abstract

**Background and Objectives:**

Periodontitis is a chronic multifactorial inflammatory disease caused by the excessive host immune response to bacterial infection, leading to periodontal tissue destruction. Owing to their plasticity, macrophages are key players in this process, and B10 cells, with their immunosuppressive efects, are vital for periodontal immunity. We propose that, in periodontitis, B10 cells transmit immunosuppressive signals *via* programmed cell death ligand-1 (PD-L1) /programmed cell death protein 1 (PD-1) signalling, stimulating macrophage diferentiation, alleviating inflammation, restoring homeostasis, and reducing alveolar bone resorption. The aim of this study was to investigate the efect of B10 cells on the polarization of macrophages in the context of periodontitis and the related molecular mechanism.

**Methods:**

B10 cells were cocultured with RAW264.7 cells in the presence or absence of a Transwell insert. The M2 macrophage proportion and PD-1 expression in macrophages were assessed by flow cytometry and quantitative polymerase chain reaction (qPCR). PD-L1 knockout (KO) B10 cells and wild-type B10 cells were subsequently cocultured with macrophages separately. For *in vivo* experiments, we injected phosphate-buffered saline (PBS), B10 cells, or PD-L1 KO B10 cells into periodontitis model mice. We evaluated outcomes *via* microcomputed tomography, histological analysis, and tartrate-resistant acid phosphatase (TRAP) staining and measured the messenger ribonucleic acid (mRNA) expression levels of tumor necrosis factor-α (TNF-α), receptor activator of nuclear factor kappa-B ligand (RANKL), interferon-γ (IFN-γ), interleukin 10 (IL-10), and osteoprotegerin (OPG) in gingival tissue surrounding the maxillary second molar *via* qPCR.

**Results:**

Compared with the indirect coculture, the direct coculture of macrophages with B10 cells led to a greater proportion of M2 macrophages and increased PD-1 expression levels in macrophages. Coculturing macrophages with PD-L1 KO B10 cells confirmed that B10 cells induced the M2 polarization of macrophages and upregulated PD-L1 expression in macrophages *via* PD-L1/PD-1 signalling. Compared with the groups injected with PBS or PD-L1 KO B10 cells, the B10 cell group exhibited significant decreases in local inflammatory factor levels, alveolar bone resorption, and the number of bone-resorbed cells within the alveolar bone area *in vivo*.

**Conclusion:**

B10 cells can regulate macrophage polarization *via* the PD-L1/PD-1 signalling pathway, thereby suppressing the inflammatory response and reducing alveolar bone resorption during periodontitis. This novel concept can guide future treatment strategies for periodontitis.

## Background

Periodontitis is one of the most prevalent chronic inflammatory diseases worldwide; it is caused by the excessive host immune response to bacterial infection and is characterized by inflammation and alveolar bone resorption.^[1]^ Dental biofilms alone cannot destroy periodontal tissue; thus, the host hyperimmune response to microorganisms constitutes the actual cause.^[2]^ Although currently employed clinical approaches to periodontitis focus on infection control,^[3]^ host immune response stabilization and bone metabolism regulation have garnered increasing attention as the understanding of the role of inflammation in periodontitis has increased. Rebalancing the immune response may be an important alternative treatment strategy for periodontitis, as the inflammatory response in periodontal tissue is orchestrated by immune cells, which modulate tissue homeostasis. This concept is supported by emerging evidence from other chronic inflammatory conditions, such as atherosclerosis, where immune-mediated inflammation plays a key role in disease progression.^[4]^

Targeted therapies for immune regulation in periodontitis, including the use of blocking antibodies, antagonists, and anti-inflammatory cytokines, have shown promising potential in inhibiting inflammatory bone resorption.^[2]^ These approaches are particularly beneficial for patients who are highly susceptible to periodontitis or systemic inflammatory diseases. As our understanding of periodontal immunity deepens, more therapeutic targets are likely to emerge, offering innovative alternative strategies for managing periodontitis.

Plasticity and flexibility are crucial macrophage characteristics,^[5]^ and macrophages can polarize into proinflammatory (M1) or anti-inflammatory (M2) phenotypes depending on their microenvironment.^[6]^ M1 macrophages mediate pathogen clearance through phagocytosis and display an inflammatory phenotype characterized by elevated expression of cluster of differentiation 80 (CD80), CD86, and CD16/32.^[7]^ Prolonged M1 macrophage activation induces excessive secretion of proinflammatory mediators, such as interleukin-1 beta (IL-1β), IL-6, tumor necrosis factor (TNF-α), and nitric oxide (NO), thereby causing chronic inflammation and tissue damage.^[8]^ Furthermore, M1 macrophages can differentiate into mature osteoclasts and promote bone resorption through various mechanisms, thus affecting alveolar bone homeostasis.^[9]^ In contrast, M2 macrophages produce anti-inflammatory cytokines, including IL-4 and IL-3, that mediate the resolution of inflammation and wound healing. Recent evidence suggests that M2 macrophages significantly modulate osteogenesis.^[10]^ Macrophage polarization is a reversible process,^[11]^ and the proportion of M2 macrophages constitutes an important disease indicator and a key therapeutic target for managing the progression of periodontitis.^[12]^

Regulatory B cells are a B-cell subset with negative regulatory effects. B10 cells are one of the most widely studied regulatory B-cell subsets and are characterized by high IL-10 levels.^[13]^ B10 cells exhibit a CD1d^hi^ CD5^+^ CD19^hi^ phenotype and constitute a mere 1-3% of the total B-cell pool, which increases to 10-20% under certain pathologic conditions.^[14]^ In a previous study, B10 cells significantly suppressed immune responses and modulated the progression of periodontal inflammation.^[15]^ Moreover, B10 cell transfer into mice effectively reduced periodontitis-induced alveolar bone resorption.^[16]^ Additionally, lipopolysaccharide (LPS)- and cytidylyl phosphate guanosine (CpG)-triggered local B10 cell induction attenuated periodontal inflammation and alveolar bone loss in mice.^[17]^

Programmed cell death protein 1 (PD-1), a cell surface receptor, and its ligand, programmed cell death ligand-1 (PD-L1), constitute a crucial activator complex for negative immunoregulatory signalling pathways. This complex plays a pivotal role in mediating peripheral immune tolerance, particularly during the effector phase of the immune response, and is instrumental in controlling autoimmunity. The significance of this pathway is underscored by the broad and well-established clinical use of immune checkpoint inhibitors targeting PD-1/PD-L1 in various diseases.^[18]^ Notably, both mouse and human macrophages express PD-1,^[19]^ and PD-L1 is upregulated in regulatory B cells in response to inflammatory signals.^[20]^

Therefore, we posit that the regulatory function of B10 cells is achieved through not only IL-10 production but also cell-cell interactions between B10 cells and macrophages. B10 cells induce M2 macrophage polarization *via* the PD-L1/PD-1 signalling pathway, promote inflammation resolution and local immune homeostasis *in vivo*, and improve periodontitis outcomes. In this study, we conducted *in vitro* experiments to validate the PD-L1/PD-1 pathway-mediated intercellular interaction between these two cell types and *in vivo* experiments using murine periodontitis models to investigate the regulatory role of B10 cell-expressed PD-L1 in inflammation.

## Methods

### Animals

Wild-type C57BL/6 mice and PD-L1 knockout (KO) C57BL/6 mice were purchased from Shanghai Model Organism. Mice aged 8-10 weeks were bred in a sterile and specific pathogen-free environment with a relative humidity of 45%-75% under a 12-h light/dark cycle. The animal procedures were approved.

### B-cell purification and induction

After the mice were euthanized in a CO^2^ chamber, their spleens were harvested, ground, and placed on a metal mesh in Iscove’s modified Dulbecco’s medium (IMDM; Gibco, MA, USA), and the resulting cell suspension was filtered through a 100-μm-mesh-size cell strainer. Then, red blood cells were lysed with ammonium-chloride-potassium (ACK) lysis buffer (Life Technologies, MA, USA), and the cell suspension was refiltered through a 40-μm-mesh-size cell strainer to obtain a single cell suspension. B cells were isolated using a magnetic column (Miltenyi Biotec, MA, USA) with a pan-B-cell isolation kit (Miltenyi Biotec, MA, USA), and splenic cell suspensions in magnetically activated cell sorting (MACS) buffer (phosphate-buffered saline [PBS]), 2 mmol/L ethylenediaminetetraacetic acid (EDTA), and 0.5% bovine serum albumin [BSA]) were prepared. Non-B cells were depleted by incubating the splenic cell suspensions with biotin-conjugated antibodies that target CD4, CD11c, CD49b, CD90, Gr-1, and Ter119. The cells were exposed to antibiotin-antibody-coupled magnetic beads (Miltenyi Biotec, MA, USA). Unlabelled cells were collected by magnetic depletion of labelled cells; the resulting purity exceeded 98.5%.

Isolated B cells were cultured for 48 h in a 6-well plate in complete medium (IMDM supplemented with 10% foetal bovine serum, 2 mmol/L L-glutamine, 100 U/mL penicillin, 100 U/mL streptomycin, 2.5 μg/mL amphotericin B, and 0.1% 2-mercaptoethanol) with or without 10 μg/mL LPS (from strain American Type Culture Collection [ATCC] 33277) and 10 μmol/L CpG (5′-TCGTCGTTTTGTCGTTTTGTCGTT-3′) to obtain B and B10 cells.

### Cell culture

The murine macrophage line RAW264.7 (ATCC TIB-71) was cultured in 6-well plates with Dulbecco’s modified Eagle’s medium (DMEM; Life Technologies, MA, USA) supplemented with 10% foetal calf serum (Atlanta Biologicals, GA, USA), 1% penicillin and streptomycin (Life Technologies, MA, USA), and 1% L-glutamine. Upon cell adhesion, RAW264.7 cells were stimulated with 100 μg/L phorbol 12-myristate 13-acetate (PMA) for 72 h.

B10 or B cells were cocultured with RAW264.7 cells in plates with or without Transwell inserts. For the two Transwell groups, complete culture medium was used, B10 or B cells were placed in the upper chamber, and adherent macrophages were placed in the lower chamber. RAW264.7 cells cultured alone served as the control group.

### Flow cytometry and cell sorting analyses

The cultured cells were washed with cell staining buffer and stained after blocking with the Fc antigen. The cells were stained with fluorescein 5-isothiocyanate (FITC)-conjugated anti-mouse CD163, phycoerythrin (PE)-conjugated anti-mouse CD279, FITC-conjugated anti-mouse CD274, PE-conjugated anti-mouse CD1d, and FITC-conjugated anti-mouse CD5 antibodies. All reagents and antibodies were purchased from BioLegend (San Diego, CA). The data were collected on a FACSAria flow cytometer (BD Biosciences, NJ, USA), and subsequent analysis was performed using FlowJo 10.7.1 (TreeStar, Inc., San Carlos, CA). CD1d^hi^ CD5^+^ B cells were sorted using a FACSAria flow cytometer in the animal experiments.

### Real-time quantitative polymerase chain reaction (qRT-PCR)

Gingival tissue surrounding the maxillary second molar was harvested and homogenized in lysis buffer using a tissue homogenizer (Omni, GA, USA). Using a PureLink ribonucleic acid (RNA) Mini Kit (Ambion, MA, USA), total RNA was extracted from both cells and gingival tissue samples. Complementary deoxyribonucleic acid (cDNA) was synthesized using a SuperScript II Reverse Transcriptase Kit (Invitrogen, MA, USA), and real-time fluorescence was used to assess the expression levels of PD-L1, PD-1, TNF-α, receptor activator of nuclear factor kappa-Β ligand (RANKL), interferon-γ (IFN-γ), IL-10, and osteoprotegerin (OPG) in these samples. Additionally, qRT-PCR was performed using LightCycler synergetic binding reagent (SYBR) Green I master mix and a LightCycler 480 instrument (Roche, Basel, Switzerland). Glyceraldehyde 3-phosphate dehydrogenase (GAPDH) served as an internal control for normalization.

### Ligature-induced periodontitis model and local administration

Wild-type mice were randomly divided into four distinct groups. Healthy mice were used as blank controls. In the remaining three groups, a ligation-induced experimental periodontitis model was established by placing a silk thread (6-0; Fisher Scientific, MA, USA) around the maxillary second molar, which was retained for 2 weeks. The day of ligation was day 0; on days 0, 3, 6, and 9 of the experimental timeline, 2 μL of PBS, B10 cells, or PD-L1 KO B10 cells were separately injected into the palatal gingiva surrounding the second molar bilaterally in the maxilla of the mice in each group. The mice were sacrificed on the 14th day, and the relevant specimens were collected for further analysis.

### Microcomputed tomography (micro-CT)

The skeletal parameters of the alveolar bone were analysed *via* micro-CT (SkyScan1172, Bruker, MA, USA). The maxillae of the mice were harvested, fixed with 4% paraformaldehyde for 24 h, and affixed to a scaffold immersed in 1 × PBS. The region of interest (ROI) was defined as the alveolar bone between the distal and mesial parts of the first and third molars, respectively. The M2 cementoenamel junction of the alveolar bone crest (CEJ-ABC) distance, bone mineral density (BMD), alveolar bone volume/total volume (BV/TV), trabecular bone thickness (Tb. Th.), trabecular bone number (Tb. N.), and trabecular separation (Tb. SP) were measured.

### Histological analysis of alveolar bone

The maxillae were fixed with 4% paraformaldehyde for 24 h. After 4 weeks of decalcification with 20% EDTA, 5-μm-thick alveolar bone sections were prepared for histological and immunostaining analyses. For haematoxylin and eosin (HE) staining, paraffin-embedded sample sections were stained with haematoxylin, dehydrated, mounted with neutral resin, and observed under a 40 × objective lens. Tartrate-resistant acid phosphatase (TRAP) staining was performed using an acid phosphatase kit (Cat. No. 378A; Sigma, MO, USA), and the number of multinucleated tartrate resistant acid phosphatase (TRAP)-positive cells on the alveolar bone surface was determined and served as an indicator of active osteoclasts.

### Statistical analysis

Statistical analysis was conducted using SPSS Statistics version 19.0 (SPSS Inc., IL, USA). The results are presented as the mean values accompanied by their respective standard deviations (SDs). Intergroup comparisons were performed using the Student’s *t* test, and statistical significance was set at *P* < 0.05.

## Results

### Direct contact with B10 cells induces M2 macrophage polarization and upregulates the expression of PD-L1 in macrophages

**Figure 1:**
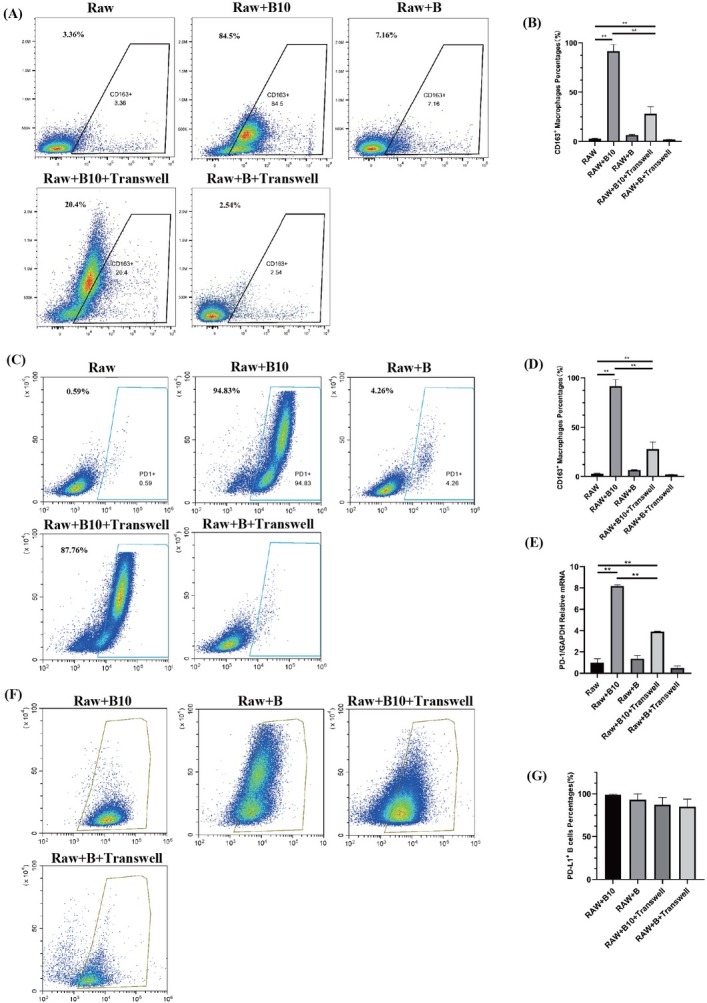
B10 cell interactions drove M2 macrophage polarization. B10 cells or B cells were cocultured with RAW264.7 cells with or without Transwell inserts for 72 h. A, B: Flow cytometric analysis of the proportion of M2 macrophages; C, D: Flow cytometric analysis of the proportion of PD-1+ macrophages; E: qRT-PCR was performed to analyse PD-1 mRNA expression in macrophages in each group; F, G: PD-L1 expression in B cells and B10 cells was tested by flow cytometric analysis. The data are expressed as the means ± SDs of triplicate wells from one experiment. Student’s *t* test (*n* = 6 animals/group); ^**^P < 0.01. PD-1: programmed cell death protein 1; PD-L1: programmed cell death ligand-1; CD: cluster of differentiation.

To investigate the impact of direct contact between B10 cells and macrophages on macrophages, we cocultured mouse B10 cells and macrophages under various conditions. Compared with those in the blank control group and the Transwell group, the proportion of M2 macrophages in the coculture group, in which macrophages were in direct contact with B10, was significantly greater (*P* < 0.01; Figure 1A, B). Thus, B10 cells can induce macrophage polarization towards the M2 phenotype. Direct contact between these two cell types significantly increased the proportion of M2 macrophages. Compared with the Transwell group, the experimental group with B10 cell coculture presented notable increases in both the proportion of PD-1^+^ macrophages and the messenger RNA (mRNA) level of PD-1 in macrophages (*P* < 0.01; Figure 1C, 1D, 1E). Thus, direct macrophage-B10 contact significantly increased PD-1 expression in macrophages. The proportion of PD-L1^+^ B10 cells was greater than that of PD-L1^+^ B cells, but the difference was not significant (*P* > 0.05; Figure 1F, 1G).

### B10 cell-triggered PD-L1 expression induces M2 macrophage polarization and upregulates the expression of PD-L1 in macrophages

**Figure 2:**
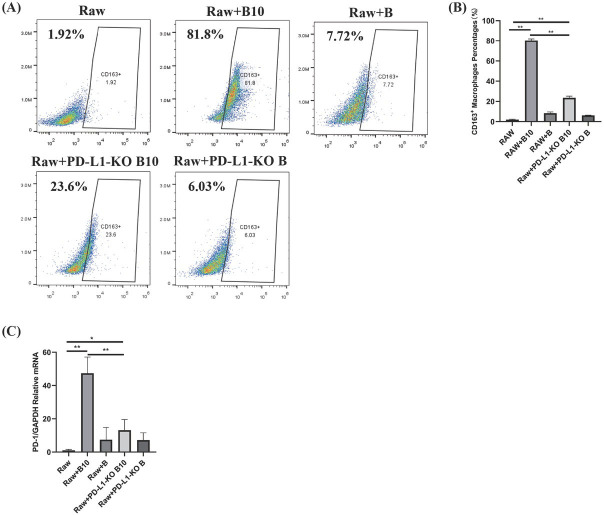
Effect of PD-L1 expressed by B10 cells on M2 macrophage polarization and PD-1 expression in macrophages. A, B: Flow cytometric analysis of M2 macrophages cocultured with B10 cells and PD-L1 KO B10 cells; C: The gene expression level of PD-1 in macrophages in the wild-type B10 cell group, PD-L1-KO B10 cell group and control group. The data are expressed as the means ± SDs of triplicate wells from one experiment. Student’s *t* test (*n* = 6 animals/group); ^*^P < 0.05; ^**^P < 0.01. PD-L1: programmed cell death ligand-1; CD: cluster of differentiation; KO: knockout.

To directly examine PD-1/PD-L1 binding between B10 cells and macrophages, we cocultured PD-L1-KO B10 cells and B10 cells separately. Flow cytometry revealed that the proportion of M2 macrophages was significantly greater in the wild-type B10 cell group than in the PD-L1-KO group (*P* < 0.05; Figure 2A, B). This finding confirms the involvement of B10 cell-expressed PD-L1 in inducing macrophage polarization towards the M2 phenotype. Furthermore, the PD-1 expression level in macrophages was significantly greater in the wild-type B10 cell group than in both the PD-L1-KO B10 cell (*P* < 0.01) and the blank control (*P* < 0.01) groups (Figure 2C). These findings indicate that PD-L1 expressed by B10 cells effectively upregulates PD-1 expression in macrophages.

### PD-L1 expressed by B10 cells reduces alveolar bone resorption in mice

**Figure 3:**
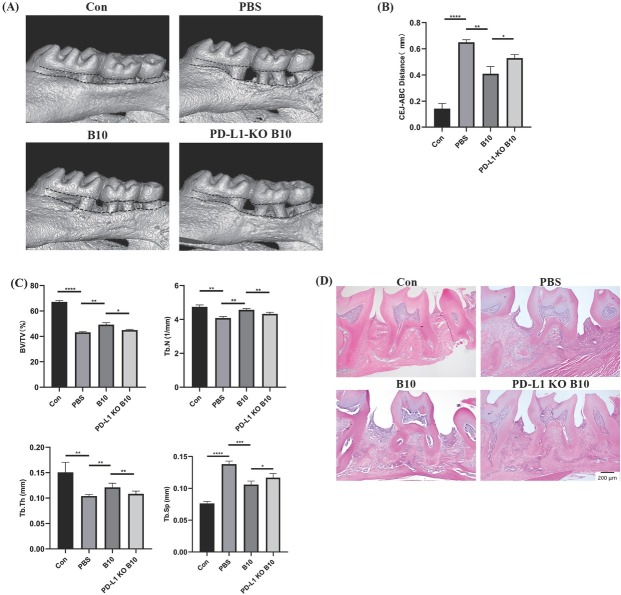
Effect of PD-L1 expressed by B10 cells on alveolar bone resorption. A group of healthy mice was used as the blank control group. Silk ligatures were tied around the maxillary secondary molars on day 0, and injections of PBS, B10 cells, and PD-L1 KO B10 cells were separately administered on days 0, 3, 6, and 9 in each group. A: Microscopy and micro-CT analysis of molars from mice with periodontitis induced by ligation; B: Periodontitis bone loss quantified by the CEJ-ABC distance on the buccal and interdental surfaces; C: Quantification of bone parameters within the ROI in the alveolar bone; D: HE staining of the periodontal tissue in the control group and ligation-induced periodontitis groups. The data are expressed as the means ± SDs. Student’s *t* test (*n* = 8 animals/group); ^*^P < 0.05; ^**^P < 0.01; ^***^P < 0.001; ^****^P < 0.0001. PD-L1: programmed cell death ligand-1; KO: knockout; PBS: phosphate-buffered saline.

After confirming the immunoregulatory effect of PD-1/PD-L1 binding between B10 cells and macrophages, we established a mouse model of periodontitis to investigate the impact of B10 cell-expressed PD-L1 on periodontitis progression and bone homeostasis. The results demonstrated that the B10 cell-injected group had a significantly greater (*P* < 0.05) level of alveolar bone resorption than the PD-L1 KO B10 cell-injected group did (Figure 3A, 3B). Additionally, BV/TV (*P* < 0.05), Tb. *N* (*P* < 0.01), and Tb. Th (*P* < 0.01) significantly decreased, whereas Tb. Sp significantly increased (*P* < 0.05; Figure 3C). Therefore, B10 cell-expressed PD-L1 can inhibit alveolar bone resorption.

HE staining revealed that the periodontal tissue in the control group exhibited normal characteristics, whereas that in all the ligation-induced periodontitis groups exhibited connective tissue inflammation and epithelial atrophy caused by ligation and loss of attachment. Additionally, there was a reduction in the height of the alveolar bone crest. The PBS group presented the most severe symptoms. Compared with the PD-L1 KO B10 group, the B10 group exhibited reduced inflammation and rather slower destruction of periodontal tissue and alveolar bone resorption (Figure 3D).

### The expression of PD-L1 by B10 cells modulates the inflammatory response in periodontal tissues

**Figure 4:**
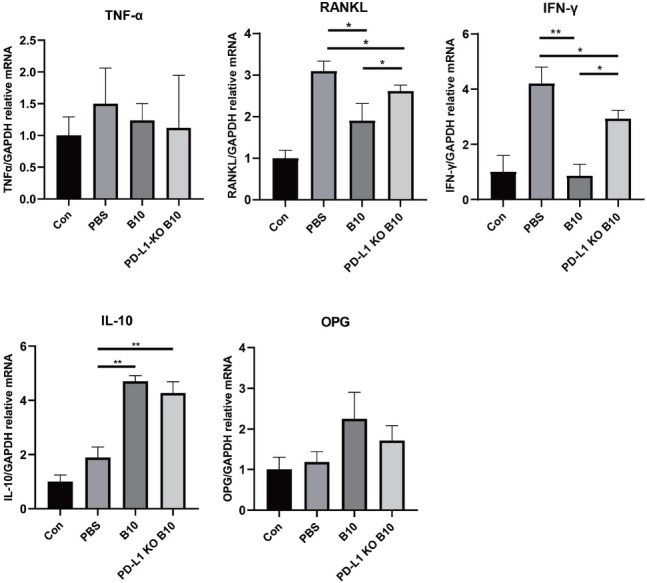
Effect of PD-L1 expressed by B10 cells on the inflammatory response of periodontal tissue. mRNA expression of TNF-α, RANKL, IFN-γ, IL-10, and OPG was measured *via* qRT-PCR. The data are expressed as the means ± SDs. Student’s *t* test (*n* = 8 animals/group); ^*^P < 0.05; ^**^P < 0.01. TNF-α: tumor necrosis factor-α; RANKL: receptor activator of nuclear factor kappa-B ligand; IFN-γ: interferon-γ; IL-10: interleukin-10; PD-L1: programmed cell death ligand-1; KO: knockout; PBS: phosphate-buffered saline.

Compared with the PBS group, the groups injected with cells presented significantly lower levels of RANKL and IFN-γ and significantly higher levels of IL-10 (both *P* < 0.05). Compared with the PD-L1-KO B10 group, the B10 group presented significantly lower levels of RANKL and IFN-γ (*P* < 0.05). No significant intergroup differences in the OPG or TNF-α mRNA levels were observed (Figure 4).

### The expression of PD-L1 by B10 cells reduces the number of osteoclasts in periodontal tissues

**Figure 5:**
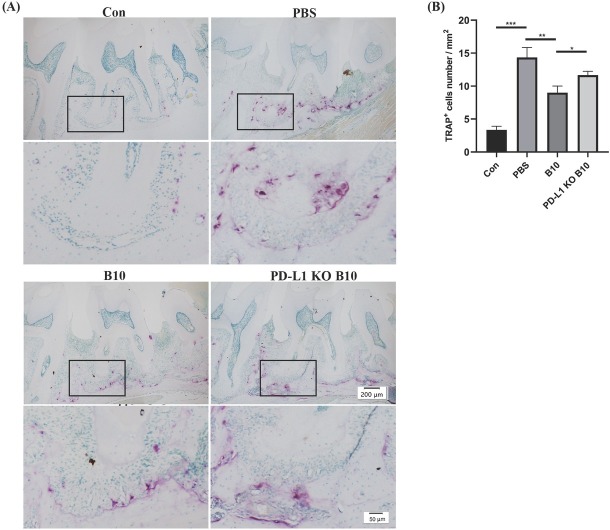
Effect of PD-L1 expressed by B10 cells on osteoclasts in periodontal tissue. a Representative images of TRAP-stained osteoclasts from a mouse model. Left to right: healthy control, PBS, B10 cell, and PD-L1-KO B10 cell groups. Row 1 Scale bar= 200 μm, row 2 Scale bar= 50 μm. B Comparison of osteoclast numbers/mm^2^ of bone surface area among the groups in (A). The data are expressed as the means ± SDs. Student’s *t* test (*n*=8 animals/group); ^*^P<0.05; ^**^P<0.01; ^***^P < 0.001.

The results revealed a significant increase in the number of multinucleated TRAP-positive cells along the alveolar bone surface after ligation (*P* < 0.01). Compared with the PD-L1 KO B10 group, the PBS group presented the greatest number of TRAP-positive cells, whereas the B10 group presented a significantly lower number of osteoclasts (*P* < 0.05; Figure 5A, 5B).

## Discussion

Currently employed clinical treatments for periodontitis mainly focus on infection control but fail to address the imbalance in the immune response and bone metabolism homeostasis. This oversight underscores the potential value of immunological interventions, which can directly mitigate the immune-mediated pathogenesis of periodontitis. Notably, B10 cells have been shown to negatively regulate periodontal inflammation and inhibit bone loss *via* IL-10 production.^[15]^ Our study highlights a direct cell-cell interaction between B10 cells and macrophages in coculture, which promotes M2 macrophage polarization and upregulates macrophage PD-1 expression. Furthermore, experiments utilizing PD-L1 KO B10 cells confirmed that macrophage polarization is mediated through the PD-L1/PD-1 signalling pathway. In a murine ligation-induced periodontitis model, local injections of PBS, B10 cells, or PD-L1 KO B10 cells demonstrated that B10 cell-mediated suppression of the PD-L1/PD-1 pathway effectively reduced periodontal inflammation and slowed alveolar bone resorption *in vivo*.

Additionally, direct coculture of B10 cells with macrophages increased the proportion of M2 macrophages. This finding supports our hypothesis that physical contact between the two cell types of triggers cell-cell interactions. Recent studies have shown that CpG-induced B10 cell activation is closely linked to PD-L1 expression. By expressing high levels of IL-10 and PD-L1, regulatory B cells can synergistically modulate immunity,^[21,22]^ Moreover, macrophages play crucial roles in PD-1/PD-L1 signalling and contribute to the immunosuppressive microenvironment.^[23]^ PD-L1-mediated immunosuppression relies on direct physical contact between cells.^[24,25]^ On this basis, we hypothesized that direct cell-cell interactions between B10 cells and macrophages involve the PD-L1/PD-1 pathway. We then analysed and found that B10 cells expressed higher levels of PD-L1 than normal B cells did. Additionally, when B10 cells and macrophages were cocultured directly, the level of PD-1 expressed by macrophages was increased. Several studies have reported that inflammatory mediators, including IL-10, induce PD-L1 production.^[26]^ For example, CpG-stimulated regulatory B cells exhibit high expression levels of PD-L1.^[27]^ Both mouse and human macrophages express PD-1, and their PD-1 levels increase with disease progression. Notably, PD-1+ cells are significantly more abundant in the M2 than in the M1 macrophage population.^[19]^ These findings are consistent with our experimental results, suggesting that B10 cells regulate macrophage polarization through the PD-L1/PD-1 pathway. This mechanism not only highlights the intricate interplay between B10 cells and macrophages but also offers a potential therapeutic target for managing periodontitis by restoring immune homeostasis and reducing inflammatory bone resorption.

To test this hypothesis, we conducted experiments utilizing PD-L1 KO mice. The results revealed the involvement of B10 cell-expressed PD-L1 in the induction of M2 macrophage polarization. This finding aligns with observations in patients with glioblastoma and melanoma, where macrophages play a pivotal role in PD-1/PD-L1 signalling, fostering an immunosuppressive microenvironment. Notably, high expression levels of PD-L1 are strongly associated with a high proportion of M2 macrophages, as evidenced by the upregulation of CD206 in macrophages in our study.^[23,28,29]^

We subsequently established a ligature-induced murine periodontitis model, which not only is convenient and reproducible but also serves as a crucial tool for exploring the immunopathological mechanisms of periodontitis.^[30]^ Commonly used methods for inducing periodontitis in mice include ligature induction, LPS injection, and oral gavage.^[31, 32, 33]^ Among these, the ligature-induced model offers the advantage of initiating periodontitis at a specific time, allowing predictable tissue destruction and enabling studies on inflammation resolution postligation. Following Abe’s method,^[30]^ we induced experimental periodontitis using ligatures. Hu *et al*. demonstrated that 14 days of ligation of the second molar induced periodontitis and alveolar bone loss. We adopted the same procedure.^[34]^ Using this ligature model, we observed that, compared with the PD-L1 KO B10 cell-treated group, the wild-type B10 cell-treated group demonstrated significantly less periodontal inflammation and alveolar bone loss. Compared with the group treated with wild type (WT) B10 cells, the group treated with PD-L1 KO B10 cells presented a greater number of TRAP-positive cells, which might further contribute to increased bone loss.

In our study, the reduced mRNA levels of the gingival inflammatory factors IFN-γ and RANKL indicated that B10 cell PD-L1 expression suppressed the inflammatory response and reduced periodontitis-associated alveolar bone loss. Notably, previous reports have demonstrated similar findings in a periodontitis model using transgenic mice overexpressing PD-L1 in gingival basal keratinocytes, where PD-L1 overexpression reduced ligation-induced inflammation in periodontal tissues and alveolar bone resorption.^[35]^ Additionally, *Por phyromonas gingivalis* upregulates PD-L1 expression but suppresses the inflammation in periodontal tissues, which is consistent with the results of our study.^[36]^

Our study demonstrated that B10 cells, through the PD-L1/PD-1 pathway, can effectively modulate immune responses and reduce alveolar bone loss. These findings extend previous research on the role of B10 cell-derived IL-10 in preventing bone loss in periodontitis, by revealing a new immunosuppressive mechanism through PD-L1/PD-1 signalling.^[16]^ These findings provide a novel perspective that complements existing strategies and may inform the development of innovative immunoregulatory therapies for periodontitis.

The gingival tissue was analysed as a whole in the *in vivo* experiments. The decrease in the inflammatory response may be partly attributed to other immune cells besides macrophages. To obtain a clearer understanding of the experimental results, further experiments investigating the effects of PD-1 KO in mouse macrophages are needed. Moreover, this study has certain limitations that should be acknowledged. Animal research contributes significantly to our understanding of human biology; however, animal models are limited in their ability to mimic human diseases. There are significant differences between the immune systems of mice and humans, and the microbiota associated with periodontitis in mice differ considerably from that associated with periodontitis in humans, which limits our findings.^[37]^

## Conclusion

In conclusion, B10 cell-expressed PD-L1 increases the PD-1 level in macrophages through intercellular interactions and induces the M2 polarization of macrophages, thereby reducing periodontal tissue inflammation and alveolar bone resorption in periodontitis model mice. Considering the importance of local host immunity in the development of periodontitis, the findings of this study serve as a reference for analysing the outcomes of periodontal tissue inflammation and provide novel therapeutic targets for managing periodontitis.
